# Marital status is associated with superior survival in patients with esophageal cancer: a Surveillance, Epidemiology, and End Results study

**DOI:** 10.18632/oncotarget.21609

**Published:** 2017-10-07

**Authors:** Lijun Du, John J. Kim, Binrui Chen, Shuwen Zhu, Ning Dai

**Affiliations:** ^1^ Department of Gastroenterology, Sir Run Run Shaw Hospital, School of Medicine, Zhejiang University, Hangzhou, China; ^2^ Division of Gastroenterology, Loma Linda University Medical Center, Loma Linda, CA, USA

**Keywords:** marital status, esophageal cancer, survival, surveillance, epidemiology

## Abstract

The impact of marital status on survival among patients with esophageal cancer has not been evaluated in the U.S. population in depth. The aim of the study was to investigate the impact of marital status on survival among patients diagnosed with esophageal cancer. The Surveillance, Epidemiology, and End Results (SEER) database was utilized to identify patients diagnosed with esophageal cancer between 1973 and 2013. Cox regression analysis was performed to evaluate for association between marital status on both cancer-specific and overall survival. Of the 69,139 patients with esophageal cancer, 35,863 (52%) had adenocarcinoma and 21,573 (31%) had distant SEER stage. At the time of diagnosis, 39,805 (57%) patients were married, 10,116 (15%) were single, 8,417 (12%) were divorced or separated, and 10,801 (16%) were widowed. Married patients had superior cancer-specific and overall survival compared to unmarried patients. Multivariate analysis demonstrated that single (adjusted hazard ratio (HR)=1.14, 95%CI 1.11-1.17; *P*<0.001), divorced or separated (HR=1.16, 95%CI 1.13-1.19; *P*<0.001), and widowed (HR=1.22, 95%CI 1.19-1.26; *P*<0.001) compared to married patients had higher risk of death from all causes. In conclusion, marital status was associated with superior survival among U.S. patients with esophageal cancer in a large population-based study.

## INTRODUCTION

Esophageal cancer is an aggressive gastrointestinal cancer [1, 2]. In the U.S., the majority of the patients present beyond early stage with a low 5-year survival of 20% [3, 4]. Furthermore, the incidence of esophageal cancer has been increasing in the U.S. due to a rise in esophageal adenocarincoma associated with obesity [5].

Married status as a surrogate marker for social support has conferred health and survival benefits across general populations as well as among patients with cancer [6, 7]. The survival benefit has been demonstrated specifically among patients with gastric, colorectal, pancreatic, ovarian, and head and neck cancers [8–12]. In a U.S. National Cancer Institute's Surveillance, Epidemiology, and End Results (SEER) cancer registry study, married patients were less likely to present with metastatic disease, more likely to receive definite therapy, and had a lower risk of cancer-specific mortality among patients with common cancers [13]. The protective effect of marriage was extended to patients with esophageal cancer. However, a previous population-based study from Sweden population showed no survival benefit of married patients among those who received surgery for esophageal cancer [14].

The effects of marital status on survival among patients with esophageal cancer are inconsistent across populations. Verifying the presence and magnitude of the impact of marital status on survival among this population can facilitate targeted interventions to improve outcome for those who are at risk. Therefore, the primary aim of the study was to examine the impact of marital status on overall and cancer-specific survival among patients diagnosed with esophageal cancer by using a large U.S. population-based SEER database.

## RESULTS

### Overall patient characteristics

During 1973 and 2013, 69,139 patients with esophageal cancer met the study inclusion criteria. The mean age of patients with esophageal cancer was 67.0±11.7 years, 52,516 (76%) were male, 35,863 (52%) had adenocarcinoma histology, and 21,573 (31%) presented at distant SEER stage (Table 1). Furthermore, 39,805 (57%) were married, 8,417 (12%) were divorced or separated, 10,116 (15%) were single, and 10,801 (16%) were widowed at the time of the diagnosis.

**Table 1:** Patient characteristics by marital status

	Total (%)	Married (%)	Non-married (%)
	N=69,139	N=39,805	N= 29,334
**Mean Age (SD)**	67.0±11.7	66.7±10.9	67.4±12.8
**Gender**			
Male	52,516 (76%)	33,560 (84%)	18,956 (65%)
Female	16,635 (24%)	6,249 (16%)	10,386 (35%)
**Race**			
White	55,730 (81%)	34,106 (86%)	21,624 (74%)
Black	9,789 (14%)	3,372 (8%)	6,417 (22%)
Others/Unknown	3,620 (5%)	2,327 (6%)	1,293 (4%)
**Histology**			
ACE	35,863 (52%)	23,307 (59%)	12,556 (43%)
SCC	33,276 (48%)	16,498 (41%)	16,778 (57%)
**Tumor Site**			
Upper third	6,368 (9%)	3,118 (8%)	3,250 (11%)
Middle third	16,492 (24%)	8,445 (21%)	8,047 (28%)
Lower third	36,489 (53%)	22,895 (58%)	13,594 (46%)
Overlapping	3,186 (5%)	1,732 (4%)	1,454 (5%)
Unspecified	6,604 (9%)	3,615 (9%)	2,989 (10%)
**SEER Stage**			
In situ	813 (1%)	496 (1%)	317 (1%)
Localized	16,871 (25%)	9,849 (25%)	7,022 (24%)
Regional	20,125 (29%)	11,979 (30%)	8,146 (28%)
Distant	21,573 (31%)	12,608 (32%)	8,965 (30%)
Unstaged	9,757 (14%)	4,873 (12%)	4,884 (17%)
**Therapy**			
Surgery/Radiation	9,890 (14%)	6,798 (17%)	3,092 (10%)
Surgery	9,933 (15%)	6,535 (16%)	3,398 (12%)
Radiation	29,077 (42%)	16,159 (41%)	12,918 (44%)
None	20,239 (29%)	10,313 (26%)	9,926 (34%)
**Mean Income, $ (SD)**	59,970±14,834	60,353±14,732	59,450±14,954

SD, standard deviation; SEER, Surveillance, Epidemiology and End Results; EAC, esophageal adenocarcinoma; SCC, squamous cell carcinoma.

On baseline characteristics, married patients were more likely to be male (84% vs. 65%, difference=19%, 95% confidence interval (CI) 19-20%), have adenocarcinoma (59% vs. 43%, difference=16%, 95%CI 15-16%), have insurance (12,196/13,319 (92%) vs. 7,225/9,822 (74%), difference=18%, 95%CI 17-19%), and receive cancer-directed therapy (74% vs. 66%, difference=8%, 95%CI 7-9%) compared to non-married patients. Furthermore, married patients had higher mean annual household income (60,353±14,732$ vs. 59,450±14,954$; difference=902$, 95%CI 679-1,126$) compared to non-married patients.

### Survival by marital status

**Figure 1:**
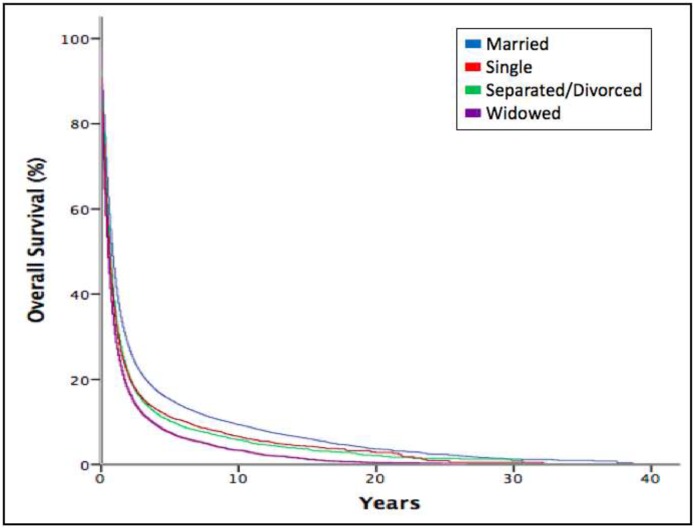
Kaplan-Meier estimates for overall survival according to marital status

**Figure 2:**
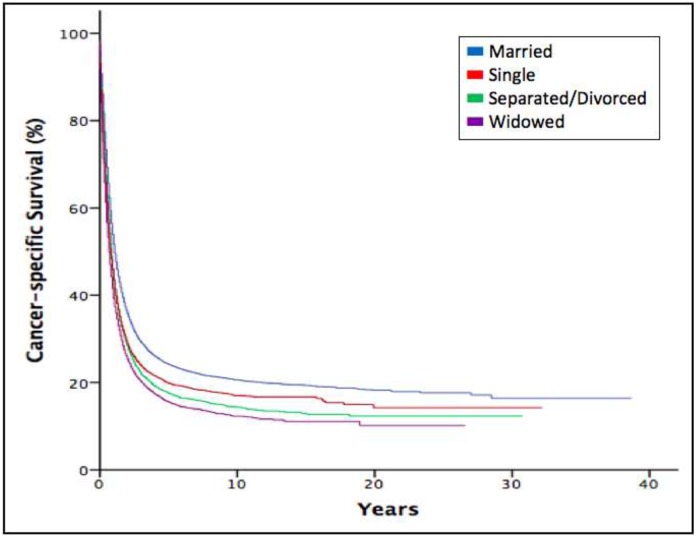
Kaplan-Meier estimates for cancer-specific survival according to marital status

We performed a Kaplan-Meier analysis to calculate overall and cancer-specific survival of esophageal cancer. For all-cause mortality analysis, the median survival for married patients was 10 months (interquartile range (IQR), 4-28), separated or divorced eight months (IQR, 3-20), single seven months (IQR, 3-19), and widowed six months (IQR, 2-16). The overall survival time among patients was different according to marital status (*P*<0.001) (Figure 1). When examining cancer-specific survival, married patients also had the longest median survival of 13 months (IQR, 5-54) compared to the other groups (*P*<0.001). The cancer-specific survival curve also favored married patients and closely mirrored the curve for overall survival (Figure 2).

### Factors associated with survival

On multivariate analyses, male gender (*P*<0.001), non-married (*P*<0.001), older patients (*P*<0.001), black race/ethnicity (*P*<0.001), squamous cell carcinoma (*P*<0.001), overlapping tumor locations (*P*<0.001), higher SEER stage (*P*<0.001), receiving no cancer-specific therapy (*P*<0.001), and lower income (*P*<0.001) were associated with poorer overall and cancer-specific survival among patients with esophageal cancer (Table 2).

**Table 2:** Multivariate Cox proportional hazards regression analysis of esophageal cancer in SEER database

Characteristics	Overall Survival HR (95% CI)	*P* value	Cancer-Specific Survival HR (95% CI)	*P* value
**Gender**				
Male	1.16 (1.14-1.18)	**<0.001**	1.13 (1.11-1.16)	**<0.001**
Female	Reference		Reference	
**Age**				
≥80	1.57 (1.52-1.61)	**<0.001**	1.45 (1.41-1.50)	**<0.001**
60-80	1.17 (1.15-1.19)	**<0.001**	1.09 (1.06-1.11)	**<0.001**
≤60	Reference		Reference	
**Race**				
Black	1.07 (1.05-1.10)	**<0.001**	1.05 (1.02-1.08)	**0.001**
White	Reference		Reference	
**Marital Status**				
Single	1.14 (1.114-1.17)	**<0.001**	1.11 (1.08-1.14)	**<0.001**
Divorced/Separated	1.16 (1.129-1.19)	**<0.001**	1.16 (1.13-1.20)	**<0.001**
Widowed	1.22 (1.194-1.26)	**<0.001**	1.21 (1.18-1.24)	**<0.001**
Married	Reference		Reference	
**Histology**				
SCC	1.20 (1.172-1.22)	**<0.001**	1.22 (1.19-1.25)	**<0.001**
ACE	Reference		Reference	
**Tumor Location**				
Overlapping	1.19 (1.139-1.25)	**<0.001**	1.28 (1.21-1.34)	**<0.001**
Lower third	1.02 (0.985-1.05)	0.310	1.04 (1.01-1.08)	**0.026**
Middle third	1.11 (1.072-1.14)	**<0.001**	1.16 (1.12-1.20)	**<0.001**
Upper third	Reference		Reference	
**SEER Stage**				
Distant	4.93 (4.48-5.42)	**<0.001**	9.81 (8.40-11.47)	**<0.001**
Regional	3.21 (2.92-3.53)	**<0.001**	6.15 (5.26-7.19)	**<0.001**
Localized	2.26 (2.06-2.49)	**<0.001**	3.94 (3.37-4.60)	**<0.001**
*In situ*	Reference		Reference	
**Therapy**				
Surgery	1.10 (1.06-1.14)	**<0.001**	1.02 (0.98-1.06)	0.289
Radiation	1.68 (1.63-1.73)	**<0.001**	1.71 (1.66-1.76)	**<0.001**
Surgery and radiation	Reference		Reference	
**Income**				
Top quintile	0.90 (0.87-0.92)	**<0.001**	0.92 (0.90-0.95)	**<0.001**
2nd quintile	0.93 (0.91-0.95)	**<0.001**	0.94 (0.91-0.96)	**<0.001**
3rd quintile	0.93 (0.91-0.95)	**<0.001**	0.93 (0.91-0.96)	**<0.001**
4th quintile	0.93 (0.91-0.96)	**<0.001**	0.94 (0.91-0.97)	**<0.001**
Bottom quintile	Reference		Reference	

HR, hazard ratio; CI, confidence interval.

Specifically for marital status, single (HR=1.14, 95%CI 1.11-1.17, *P*<0.001), separated or divorced (HR=1.16, 95%CI 1.13-1.19, *P*<0.001), and widowed (HR=1.22, 95%CI 1.19-1.26, *P*<0.001) patients had increased risk of all-cause mortality compared to married patients. On multivariate analysis evaluating for cancer-specific survival, single (HR=1.11, 95%CI 1.08-1.14, *P*<0.001), separated or divorced (HR=1.16, 95%CI 1.13-1.20, *P*<0.001), and widowed (HR=1.21, 95%CI 1.18-1.24, *P*<0.001) patients also had increased risk of cancer-specific death compared to married patients.

### Subgroup analyses for evaluating impact of marital status on survival

We further explored the associations between marital status and survival stratified by age, gender, race/ethnicity, household income, histology, tumor site, SEER stages, therapy, and insurance status (Supplementary Tables 1-9). For males, non-married patients had higher risk of death compared to married patients for overall (HR=1.18, 95%CI 1.16-1.21) and cancer-specific (HR=1.16, 95%CI 1.14-1.19) survival. For females, non-married patient had higher risk of death compared to married patients for overall (HR=1.13, 95%CI 1.09-1.17) and cancer-specific (HR=1.13, 95%CI 1.09-1.18) survival. Furthermore, non-married male patients had higher risk of all-cause (HR=1.15, 95%CI 1.12-1.18) and cancer-specific mortality (HR=1.12, 95%CI 1.08-1.15) compared to non-married female patients.

In all other subgroup analyses excluding patients without insurance, married patients also had lower risk of death compared to non-married patients for overall survival. In all other subgroup analyses excluding patients with i*n situ* cancer stage, married patients also had lower risk of death compared to non-married patients for cancer-specific survival.

## DISCUSSION

In a large population-based U.S. study of patients with esophageal cancer, married patients demonstrated superior overall and cancer-specific survival compared to those that were not married. Furthermore, among those who were not married, widowed patients had the highest risk of death followed by divorced or separated, single, and married patients after adjusting for confounding factors. Finally, male gender, older age, black race/ethnicity, squamous cell carcinoma histology, overlapping tumor locations, receiving no cancer-directed therapy, and lower income were also associated with worse survival.

Our findings are consistent with a recent U.S. study that demonstrated the protective effect of marriage among patients with cancer including those with esophageal cancer [13]. However our results are different from prior Swedish studies that demonstrated no survival benefit among patients with esophageal cancer undergoing surgery [14]. A possible reason for the inconsistent findings may be related to the difference in the study design that selected for patients who were candidates for surgery in the Swedish study. For example, a higher proportion of patients in our study had squamous cell carcinoma (29,334/69,139 (48%) vs. 149/606 (25%); difference=18%, 95%CI 14%-21%) and metastatic disease (21,573/69,139 (31%) vs. 69/606 (11%); difference=20%, 95%CI 17-22%) compared to the Swedish cohort undergoing surgery. Only 19,823 (29%) patients in our study received esophageal cancer surgery.

Physical, psychological, and sociological reasons have been proposed to explain the existence of health inequality between married and unmarried patients with cancer [15]. Sicker patients including those with advanced cancer are less likely to marry and have a higher risk of dissolution of marriage [16]. Furthermore, marriage as a surrogate indicator for social support promotes healthy lifestyle decisions and utilization of healthcare resources that may lead to early detection of cancer [17–21]. However, given that the survival benefit of married patients was observed in all cancer stages including those with metastatic disease in our study, the impact of marriage appears to extend beyond the benefit of early diagnosis. For example, our study demonstrated that married patients were more likely to receive cancer-specific therapy compared to those who were not married (74% vs. 66%, difference=8%, 95%CI 7-9%). Other studies have demonstrated higher adherence with prescribed therapy in married patients with chronic disease compared to unmarried patients which may impact outcome [22].

Esophageal cancer has unique phenotypic features which may require high level of social support for optimal care. First, patients with esophageal cancer have relatively poor prognosis with a minority of patients being candidates for curative therapy. As a result, majority of patients with esophageal cancer die within a year of diagnosis and undergo palliative care [23]. Furthermore, progressive solid food dysphagia as the primary symptom of esophageal cancer resulting from luminal tumor obstruction leave these patients particularly susceptible to malnutrition compounding the weight loss associated with cancer biology and chemoradiation treatment toxicities [24]. Previous studies have demonstrated that improved nutrition was associated with better outcomes among patients receiving treatment for esophageal cancer [25]. Therefore, many patients with advanced esophageal cancer with impaired nutrition frequently receive complex enteral or parental nutritional therapies that require social support [26]. Finally, esophageal cancer has a strong male preponderance. Our subgroup analysis as well as other studies have suggested that male patients may benefit more from social support through marriage compared to female patients with cancer.

Our study has limitations. SEER database lacked detailed individual patient information, such as financial status, medical comorbidities (i.e. mood disorders), and other forms of family support structure (e.g. children), which may potentially confound the outcome analysis. In addition, as the data on marital status was collected at the time of diagnosis, potential change in marital status as a result of esophageal cancer diagnosis was not assessed.

In summary, a large population-based U.S. study of patients with esophageal cancer demonstrated that married patients have superior overall and esophageal cancer-specific survival compared to unmarried patients. The impact of marital status on cancer survival highlights the importance of social support in the care of patients with esophageal cancer. Recognition of patients at risk may facilitate targeted support mechanisms to improve outcomes in this population.

## MATERIALS AND METHODS

### Patient population and study design

Data was obtained from the SEER program of the National Cancer Institute, which collects and publishes cancer incidence, treatment, and survival data from cancer registries covering approximately 28% of the U.S. population [3]. Morphology codes of C15.0-C15.9 and D00.1 were used to identify patient diagnosed with esophageal cancer between 1973 and 2013. Only histological codes according to ICD-O-3 for adenocarcinoma (code: 8140-8145, 8147, 8150, 8210-8211, 8255-8323, 8480-8490, 8560-8576) and squamous cell carcinoma (code: 8052-8078, 8083-8084, 8094) of the esophagus were included in the analysis. Patients were excluded if age at diagnosis was less than 18 years, had incomplete data of marital status, or the cause or time of death was unknown. Other factors including gender, race/ethnicity, marital status, histology, tumor location, SEER stages, cancer-specific therapy, household income, and insurance status were extracted from the database.

The primary endpoints were overall survival and cancer-specific survival of patients with esophageal cancer. Marital status was categorized as either married or not married at the time of diagnosis. Those that were not married were further categorized as single, separated or divorced, and widowed for the outcome analysis. Patients who reported to cohabitate with an unmarried, domestic partner (same gender, opposite gender, or unregistered) were excluded from the outcome analysis given the very low number (0.05%) of patients. Cancer stage at presentation was classified as *in situ*, localized, regional, or distant according to American Joint Committee on Cancer staging reported by SEER. Household income was derived from the patient's county median household income and stratified into quintiles for analysis. Insurance status was defined by patients who had commercial or Medicare insurance. We obtained permission to access the research data files with the reference number 13113-Nov2015. The study was approved by the review board of Sir Run Run Shaw Hospital, School of Medicine, Zhejiang University.

### Statistical analysis

SEER^*^Stat software (Version 8.2.1) was used to identify patients who met the inclusion criteria. Continuous variables were expressed as mean and standard deviations (SD) or median with IQR. Categorical values were expressed as frequency (percentages) with 95% CI. The associations between marital status and clinical characteristics were analyzed using the chi-square test or student t-test. Factors associated with survival outcomes were evaluated by Kaplan-Meier analysis and Cox regression models. Insurance status as a factor was evaluated only as a subgroup analysis given that the data was not available in the SEER database prior to the year 2007. For the survival analysis with overall survival as an outcome variable, deaths of any cause were treated as events while patients who were alive at last follow-up were censored. For the cancer specific survival analysis, deaths attributed to esophageal cancer were treated as events, while other causes of deaths other than esophageal cancer or survivors were censored. All analyses were performed with SPSS (Version 22.0). A two-sided *P*<0.05 was considered statistical significance.

## SUPPLEMENTARY MATERIALS TABLES



## Abbreviations

SEER: Surveillance Epidemiology and End Results.
