# Supplementary material for: Marital status is associated with superior survival in patients with esophageal cancer: a Surveillance, Epidemiology, and End Results study
Source: Oncotarget. 2017 Oct 7;8(56):95965–72. doi: 10.18632/oncotarget.21609 (PMC5707073; doi:10.18632/oncotarget.21609)
Supplement: Supplementary file 1 [file oncotarget-08-95965-s001.pdf]

Marital status is associated with superior survival in patients with esophageal cancer: a surveillance, epidemiology, and end results study

SUPPLEMENTARY MATERIALS

Supplementary Table 1: Multivariate analysis of marital status on overall and esophageal cancer-caused specific survival based on different age stratifications

Characteristics	Overall Survival HR (95% CI)	*P* value	Cancer-specific Survival HR (95% CI)	*P* value
**≤60**				
Non-married	1.17 (1.14-1.21)	**<0.001**	1.15 (1.11-1.19)	**<0.001**
Married	Reference		Reference	
**60-80**				
Non-married	1.17 (1.14-1.19)	**<0.001**	1.15 (1.12-1.18)	**<0.001**
Married	Reference		Reference	
**≥80**				
Non-married	1.17 (1.12-1.22)	**<0.001**	1.161 (1.10-1.22)	**<0.001**
Married	Reference		Reference	

Adjusting for gender, race/ethnicity, household income, histology, tumor site, SEER stages, therapy.

Supplementary Table 2: Multivariate analysis of marital status on overall and esophageal cancer-caused specific survival based on different gender

Characteristics	Overall Survival HR (95% CI)	*P* value	Cancer-specific Survival HR (95% CI)	*P* value
**Male**				
Non-married	1.18 (1.16-1.21)	**<0.001**	1.16 (1.14-1.19)	**<0.001**
Married	Reference		Reference	
**Female**				
Non-married	1.13 (1.09-1.17)	**<0.001**	1.13 (1.09-1.18)	**<0.001**
Married	Reference		Reference	

Adjusting for age, race/ethnicity, household income, histology, tumor site, SEER stages, therapy.

Supplementary Table 3: Multivariate analysis of marital status on overall and esophageal cancer-caused specific survival based on race/ethnicity diversity

Characteristics	Overall Survival HR (95% CI)	*P* value	Cancer-specific Survival HR (95% CI)	*P* value
**White**				
Non-married	1.17 (1.15-1.19)	**<0.001**	1.16 (1.13-1.19)	**<0.001**
Married	Reference		Reference	
**Black**				
Non-married	1.17 (1.12-1.23)	**<0.001**	1.13 (1.07-1.18)	**<0.001**
Married	Reference		Reference	
**Others/Unknown**				
Non-married	1.20 (1.12-1.30)	**<0.001**	1.22 (1.12-1.33)	**<0.001**
Married	Reference		Reference	

Adjusting for age, gender, household income, histology, tumor site, SEER stages, therapy.

Supplementary Table 4: Multivariate analysis of marital status on overall and esophageal cancer-caused specific survival based on household income

Characteristics	Overall Survival HR (95% CI)	*P* value	Cancer-specific Survival HR (95% CI)	*P* value
**Top quintile**				
Non-married	1.19 (1.14-1.23)	**<0.001**	1.17 (1.12-1.22)	**<0.001**
Married	Reference		Reference	
**2nd quintile**				
Non-married	1.19 (1.14-1.23)	**<0.001**	1.19 (1.13-1.24)	**<0.001**
Married	Reference		Reference	
**3rd quintile**				
Non-married	1.17 (1.12-1.21)	**<0.001**	1.14 (1.10-1.19)	**<0.001**
Married	Reference		Reference	
**4th quintile**				
Non-married	1.16 (1.12-1.21)	**<0.001**	1.16 (1.11-1.21)	**<0.001**
Married	Reference		Reference	
**Bottom quintile**				
Non-married	1.17 (1.13-1.22)	**<0.001**	1.15 (1.10-1.21)	**<0.001**
Married	Reference		Reference	

Adjusting for age, gender, race/ethnicity, histology, tumor site, SEER stages, therapy.

Supplementary Table 5: Multivariate analysis of marital status on overall and esophageal cancer-caused specific survival based on different histotypes

Characteristics	Overall Survival HR (95% CI)	*P* value	Cancer-specific Survival HR (95% CI)	*P* value
**ACE**				
Non-married	1.19 (1.16-1.22)	**<0.001**	1.18 (1.15-1.22)	**<0.001**
Married	Reference		Reference	
**SCC**				
Non-married	1.16 (1.13-1.19)	**<0.001**	1.14 (1.11-1.17)	**<0.001**
Married	Reference		Reference	

Adjusting for age, gender, race/ethnicity, household income, tumor site, SEER stages, therapy.

Supplementary Table 6: Multivariate analysis of marital status on overall and esophageal cancer-caused specific survival based on different tumor sites

Characteristics	Overall Survival HR (95% CI)	*P* value	Cancer-specific Survival HR (95% CI)	*P* value
**Upper third**				
Non-married	1.12 (1.06-1.17)	**<0.001**	1.11 (1.04-1.18)	**0.001**
Married	Reference		Reference	
**Middle third**				
Non-married	1.17 (1.13-1.21)	**<0.001**	1.15 (1.11-1.20)	**<0.001**
Married	Reference		Reference	
**Lower third**				
Non-married	1.18 (1.16-1.21)	**<0.001**	1.17 (1.14-1.20)	**<0.001**
Married	Reference		Reference	
**Overlapping**				
Non-married	1.14 (1.05-1.23)	**0.001**	1.106 (1.02-1.21)	**0.021**
Married	Reference		Reference	

Adjusting for age, gender, race/ethnicity, household income, histology, SEER stages, therapy.

Supplementary Table 7: Multivariate analysis of marital status on overall and esophageal cancer-caused specific survival based on different cancer stages

Characteristics	Overall Survival HR (95% CI)	*P* value	Cancer-specific Survival HR (95% CI)	*P* value
**In situ**				
Non-married	1.30 (1.06-1.59)	**0.011**	1.24 (0.88-1.73)	0.222
Married	Reference		Reference	
**Localized**				
Non-married	1.19(1.15-1.24)	**<0.001**	1.17 (1.12-1.22)	**<0.001**
Married	Reference		Reference	
**Regional**				
Non-married	1.15 (1.11-1.18)	**<0.001**	1.12 (1.08-1.17)	**<0.001**
Married	Reference		Reference	
**Distant**				
Non-married	1.17 (1.13-1.20)	**<0.001**	1.16 (1.12-1.20)	**<0.001**
Married	Reference		Reference	

Adjusting for age, gender, race/ethnicity, household income, histology, tumor site, therapy.

Supplementary Table 8: Multivariate analysis of marital status on overall and esophageal cancer-caused specific survival based on different therapies

Characteristics	Overall Survival HR (95% CI)	*P* value	Cancer-specific Survival HR (95% CI)	*P* value
**Radiation and surgery**				
Non-married	1.11 (1.05-1.17)	**<0.001**	1.09(1.03-1.16)	**0.004**
Married	Reference		Reference	
**Radiation**				
Non-married	1.19 (1.13-1.25)	**<0.001**	1.12 (1.05-1.19)	**0.001**
Married	Reference		Reference	
**Surgery**				
Non-married	1.13 (1.10-1.16)	**<0.001**	1.11 (1.08-1.14)	**<0.001**
Married	Reference		Reference	
**None**				
Non-married	1.21 (1.17-1.24)	**<0.001**	1.22 (1.18-1.27)	**<0.001**
Married	Reference		Reference	

Adjusting for age, gender, race/ethnicity, household income, histology, tumor site, and SEER stage.

Supplementary Table 9: Multivariate analysis of marital status on overall and esophageal cancer-caused specific survival based on insurance

Characteristics	Overall Survival HR (95% CI)	*P* value	Cancer-specific Survival HR (95% CI)	*P* value
**Insured**				
Non-married	1.19 (1.15-1.24)	**<0.001**	1.19 (1.14-1.24)	**<0.001**
Married	Reference		Reference	
**Uninsured**				
Non-married	1.08 (0.99-1.17)	0.076	1.13 (1.03-1.25)	**0.012**
Married	Reference		Reference	

Adjusting for age, gender, race/ethnicity, household income, histology, tumor site, SEER stage and therapy.
